# GSHSite: Exploiting an Iteratively Statistical Method to Identify S-Glutathionylation Sites with Substrate Specificity

**DOI:** 10.1371/journal.pone.0118752

**Published:** 2015-04-07

**Authors:** Yi-Ju Chen, Cheng-Tsung Lu, Kai-Yao Huang, Hsin-Yi Wu, Yu-Ju Chen, Tzong-Yi Lee

**Affiliations:** 1 Institute of Chemistry, Academia Sinica, Taipei, Taiwan; 2 Department of Computer Science and Engineering, Yuan Ze University, Taoyuan, Taiwan; 3 Innovation Center for Big Data and Digital Convergence, Yuan Ze University, Taoyuan, Taiwan; International Centre for Genetic Engineering and Biotechnology (ICGEB), INDIA

## Abstract

*S*-glutathionylation, the covalent attachment of a glutathione (GSH) to the sulfur atom of cysteine, is a selective and reversible protein post-translational modification (PTM) that regulates protein activity, localization, and stability. Despite its implication in the regulation of protein functions and cell signaling, the substrate specificity of cysteine *S*-glutathionylation remains unknown. Based on a total of 1783 experimentally identified *S*-glutathionylation sites from mouse macrophages, this work presents an informatics investigation on *S*-glutathionylation sites including structural factors such as the flanking amino acids composition and the accessible surface area (ASA). TwoSampleLogo presents that positively charged amino acids flanking the *S*-glutathionylated cysteine may influence the formation of *S*-glutathionylation in closed three-dimensional environment. A statistical method is further applied to iteratively detect the conserved substrate motifs with statistical significance. Support vector machine (SVM) is then applied to generate predictive model considering the substrate motifs. According to five-fold cross-validation, the SVMs trained with substrate motifs could achieve an enhanced sensitivity, specificity, and accuracy, and provides a promising performance in an independent test set. The effectiveness of the proposed method is demonstrated by the correct identification of previously reported *S*-glutathionylation sites of mouse thioredoxin (TXN) and human protein tyrosine phosphatase 1b (PTP1B). Finally, the constructed models are adopted to implement an effective web-based tool, named GSHSite (http://csb.cse.yzu.edu.tw/GSHSite/), for identifying uncharacterized GSH substrate sites on the protein sequences.

## Introduction

*S*-glutathionylation is a redox-dependent PTM that involves the covalent attachment of glutathione (GSH) to the thiol group of cysteine residues and regulates by physiological GSH level and GSH/GSSG ratio through spontaneous or enzymatic reaction [1–5]. In addition to regulating redox signaling, *S*-glutathionylation also serves to modulate cancer migration, cell death and survival, energy metabolism and glycolysis, as well as protein folding and degradation from bacteria to human [2]. The various targets of *S*-glutathionylation also participate in pathogenesis of many diseases, such as neurodegenerative diseases, metabolic disorders and cancers [6,7]. Due to the labile nature and low abundance of *in vivo S*-glutathionylation, the detail characteristics and mechanisms of *S*-glutathionylation still await to be clarified. To our knowledge, the protein database of human, mouse, or rat possess only consist of approximate 2% cysteine residues. However, only cysteine residue containing lower pKa in a basic environment perhaps in close three-dimensional proximity to Arg, Lys, or His residues is more accessible by GSH modification [8].

To date, numerous methods have been directed toward mass spectrometry-based *S*-glutathionylomics using various biological systems to investigate and identify more than thousands of *S*-glutathionylated targets and sites [9–11]. As for the growing number of experimentally-identified *S*-glutathionylated peptides, a curated database is of urgent need to facilitate further biological investigation of *S*-glutathionylated proteins and the substrate specificities of *S*-glutathionylation sites. Bioinformatic approaches are powerful tools for prediction of the susceptibility of individual cysteine residues to *S*-glutathionylation. Although several algorithms and public servers have been developed to analyze and predict the reactive state of cysteine [12,13] and oxidative yet disulfide cysteines [14,15], few specific information of *S*-glutathionylation targets and sites is reported. Sun et al. have computationally identified *S*-glutathionylation motifs by functional annotation and *S*-glutathionylation sites prediction by collecting 43 experimentally *S*-glutathionylated proteins and 227 corresponding sites [16]. Other potential novel consensus *S*-glutathionylation motifs and substrate site specificities remains unclear.

To further investigate potential *S*-glutathionylation motifs in primary amino acid sequence, the *in silico* characterization, i.e. amino acid composition (AAC) and accessible surface area (ASA), of protein *S*-glutathionylation sites is applied to distinguish the *S*-glutathionylation sites *versus* non-*S*-glutathionylation sites. In this study, we anticipate to characterize the *S*-glutathionylation sites with the consideration of substrate specificity of GSH. This study presents a statistical method for identifying *S*-glutathionylation sites and potential consensus motifs by maximal dependence decomposition (MDD) [17]. With the application of MDD, a large group of aligned sequences can be moderated into subgroups that capture the most significant dependencies between positions. By further evaluation using five-fold cross-validation, the support vector machine (SVM) models trained with MDD-clustered subgroups could improve predictive accuracy when compared to the model without MDD clustering. Moreover, the experimental *S*-glutathionylation data from published database (independent set) are used to test the effectiveness of the models in cross-validation. To facilitate the study of protein *S*-glutathionylation, the identified substrate motifs were exploited to implement a web-based resource for identifying *S*-glutathionylation sites with potential motifs.

## Materials and Methods

### Data collection and preprocessing of training set and independent test set

With the MS-based high-throughput *S*-glutathionylomic data, the experimentally verified *S*-glutathionylated cysteines from mouse macrophages [18] constituted the positive data of training set, and non-*S*-glutathionylated cysteines on these *S*-glutathionylated proteins were used as the negative data. As shown in Table 1, 1783 positive and 8423 negative data on 1005 *S*-glutathionylated proteins were obtained. In order to avoid a biased prediction performance for a binary classification between positive and negative data, the negative training data was balanced with the positive training data. A *K*-means clustering method based on sequence identity [19,20] was employed for acquiring a subset that represented the whole negative data set. The number of corresponding positive data was set as the value of *K*, which denoted the number of samples obtained from the negative set. This resulted in an equal number of positive and negative sequence fragments from the training data (Table 1).

**Table 1:** Data statistics of training set and independent testing set.

**Species**	**S-glutathionylation sites (Positive data)**	**Non-S- glutathionylation sites (Negative data)**
***Training set***		
Su et al., 2014 (PMID: 24333276)		
Mouse	1783	8423
***Independent testing set***		
**RedoxDB**		
Mouse	20	186
Other	222	887
**SGDB**		
Mouse	4	62
Other	71	327
**Combined non-redundant database**		
Mouse	19	213
Other	254	1054

For independent testing set, the experimentally verified *S*-glutathionylation sites were mainly extracted from RedoxDB [14] and SGDB [16]. A total of 20 *S*-glutathionylated cysteines on 11 proteins from mouse, extracted from RedoxDB were used as the positive data set, while the remaining 186 non-*S*-glutathionylated cysteines on these proteins functioned as the negative data set. SGDB contributes 4 *S*-glutathionylated cysteines on 4 proteins from mouse (positive data set), while other 62 non-*S*-glutathionylated cysteines were used as the negative data set. This study focused on the sequence-based analysis of substrate specificity of cysteine *S*-glutathionylation. The ability to distinguish the *S*-glutathionylated cysteine from the non-*S*-glutathionylated cysteine of the identified motifs would be evaluated based on cross-validation.

After the cross-validation of training set, the model with highest accuracy was further evaluated by using an independent test set. However, the positive data of independent test set may include the sequences that were homologous to training data. As for classification, the prediction performance of the trained models may be overestimated owing to the over-fitting of a training set. To this, the homologous sequences between training set and independent test set were removed. With reference to the reduction of the homology of the training set in MASA [19], two *S*-glutathionylated protein sequences with more than 30% identity were defined as homologous sequences. Two homologous sequences were specified to re-align the fragment sequences using a window length of 2*n*+1, centered on the *S*-glutathionylation sites using BL2SEQ [21]. For two fragment sequences with 100% identity, only one *S*-glutathionylation site on homologue fragment sequence in training set was kept while the other in testing set was discarded. Redundancy was removed by retaining only one record in the event of finding multiple records of the same site position and accession number. The non-redundant negative data were generated using the same approach as positive one. After the removal of redundant data, 254 positive sequence fragments and 1054 negative sequence fragments with cysteine residues were obtained for independent testing.

### Features investigation

Aside from the composition of flanking amino acids (AA), the **accessible surface areas** (ASA) around the *S*-glutathionylation sites were also investigated. Amino acid sequences with a cysteine in the center were individually extracted from positive and negative training sets using a window of length 2*n*+1 centered on substrate sites, where *n* was set to ten in this study. An orthogonal binary coding scheme was adopted to transform amino acids into numeric vectors, in the so-called 20-dimensional binary coding. For example, glycine was encoded as “10000000000000000000;” alanine was encoded as “01000000000000000000,” and so on. The number of feature vectors represented the flanking amino acids surrounding the *S*-glutathionylation site was (2*n*+1) × 20. A total of *p* vectors {*x*_*i*_, *i* = 1,. . ., *p*} were used to represent all *p* sequence fragments in the training data. Each vector in positive or negative cysteines was labeled with the class of its corresponding protein (e.g. positive or negative). For the composition of 20 amino acids surrounding the *S*-glutathionylation sites, the vector *x*_*i*_ had 20 elements for the amino acid composition (AAC) and 441 elements for the amino acid pair composition (AAPC). The 20 elements were defined as the occurrence frequencies of 20 amino acids in a sequence fragment, and the 400 elements were defined as the occurrence frequencies of 400 amino acid pairs in a sequence fragment. When the fragment sequences at N- or C-terminus are less than 21-mer, non-existing residues were filled with “X” in the corresponding position. A total of 21 types of amino acids and 441 types of amino acid pairs were presented in our setting. Using the BLOcks SUbstitution Matrix (BLOSUM62) matrix [22], the given substitution scores were derived from the alignments of amino acid sequences that had no more than 62% identity between two peptide sequences with 21 amino acids. S1 Fig. displays in detail how to generate the 441 AAPC combining BLOSUM62 features for each sequence fragment.

Refer to the method of SulfoSite [23], the positional weighted matrix (PWM) of amino acids around the *S*-glutathionylated cysteines was determined using non-homologous training data. The PWM specified the relative frequency of amino acids that surrounded the *S*-glutathionylation sites, and was utilized in encoding the fragment sequences. A matrix of *m* × *w* elements was used to represent each residue of a training dataset, where *w* stands for the window size and *m* consists of 21 elements including 20 types of amino acids and one for terminal signal. In addition, WebLogo [24,25] was adopted to generate the graphical sequence logo for the relative frequency of the corresponding amino acid at each position around the *S*-glutathionylation sites.

In the viewpoint of structural environment, several amino acid residues of a protein can be mutated without changing its structure, and two proteins may have similar structures with different amino acid compositions. Position Specific Scoring Matrix (PSSM) profiles, which have been extensively utilized in protein secondary structure prediction, subcellular localization and other bioinformatics problems [20,26–28], are adopted herein with significant improvement. The PSSM profiles were obtained by PSI-BLAST [29] against non-redundant sequences of *S*-glutathionylation sites. The matrix of (2*n*+1)×20 elements had rows centered on substrate site, extracted from the PSSM profile, where 2*n*+1 represented the window size and 20 represented the position specific scores for each type of amino acid. After that, the (2*n*+1)×20 matrix was transformed into a 20×20 matrix by summing up the rows that were associated with the same type of amino acid. Finally, every element in 20×20 matrix was divided by the window length 2*n*+1 and then normalized using the formula: $\frac{1}{1+e^{-x}}$.

A side-chain of amino acid that undergoes post-translational modification prefers to be accessible on the surface of a protein [30]. Thus, the solvent-**accessible surface area** (ASA) was used to evaluate the characteristics of *S*-glutathionylation sites. Since most of the experimental *S*-glutathionylated proteins did not have corresponding protein tertiary structures in PDB [31], an effective tool, RVP-Net [32,33], was applied to compute the ASA value from the protein sequence. RVP-net applied a neutral network to predict the real ASA of residues based on information about their neighborhood. The measurement was with a mean absolute error of 18.0–19.5%, which was defined as the absolute difference between the predicted and experimental values of relative ASA per residue [33]. The computed ASA was the percentage of the solvent-accessible area of each amino acid on the protein. The full-length protein sequences with experimentally identified *S*-glutathionylation sites were inputted to RVP-Net to compute the ASA value of all of the residues. The ASA values of amino acids around the *S*-glutathionylation sites were extracted and normalized to be between zero and one.

### Data clustering by maximal dependence decomposition

**Fig 1:**
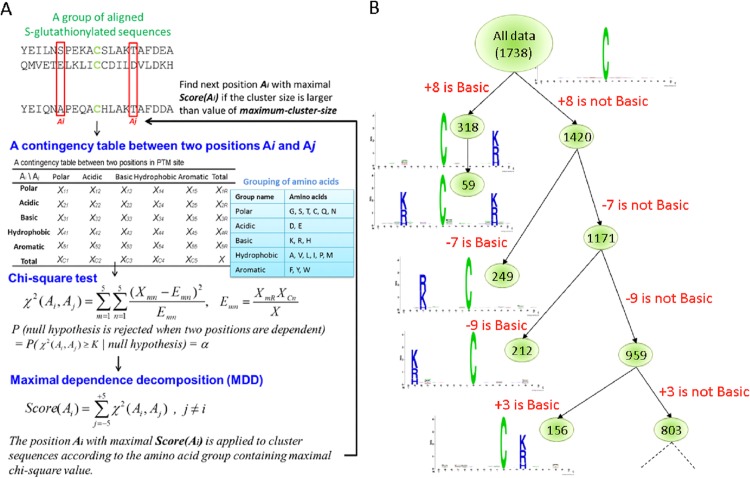
The analytical flowchart of MDDLogo application. (A) Using chi-square test to detect the maximal dependence of position, and (B) Tree-like visualization of MDDLogo-clustering result.

The aim of this study was to investigate the motifs of *S*-glutathionylation sites based on the amino acid sequences. Due to the difficulty of detecting the conserved motifs for the sequence data with a larger size, this work applied maximal dependence decomposition (MDD) [17] to cluster all sequences of *S*-glutathionylation site into subgroups, which had statistically obvious motifs. MDDLogo has been reported that the grouping of protein sequences into smaller groups is prior to computationally identifying PTM sites [34–40]. As illustrated in Fig 1A, the sequence fragments of GSH sites are extracted using a window of length 2*n*+1. Since *n* is set to 10, the position *Ai* is defined from the range of -10 to +10. Then, a contingency table of the amino acids occurrence between two positions *Ai* and *Aj* is generated. In order to extract the motifs that had conserved biochemical property of amino acids, the 20 types of amino acids were categorized into five groups, including polar, acidic, basic, hydrophobic, and aromatic groups (S1 Table). Next, MDDLogo adopted chi-square test *χ* ^2^(A_i_, A_j_) to evaluate the dependence of amino acid occurrence between two positions *Ai* and *Aj* surrounding the GSH sites. The chi-square test was defined as:

$$\chi^{\text{2}}{\text{(A}}_{\text{i}}{\text{,A}}_{\text{j}}\text{)}=\sum_{m=1}^{5}\sum_{n=1}^{5}\frac{{\left(X_{mn}-E_{mn}\right)}^{2}}{E_{mn}}$$ (1)

where X_mn_ represented the number of sequences that had the amino acids of group m in position A_i_ and had the amino acids of group n in position A_j_, for each pair (*A*_i_, *A*_*j*_) with i≠j. E_mn_ was calculated as $\frac{X_{mR}\cdot X_{Cn}}{X}$, where X_mR_ = X_m1_+ …+X_m5_, X_Cn_ = X_1n_+ …+X_5n_, and X denoted the total number of sequences. If a strong dependence were detected (defined as a X^2^ value was larger than 34.3, corresponding to a cutoff level of P = 0.01 with 16 degrees of freedom) between two positions, it will be proceeded as described by Burge and Karlin [17]. A dependence value for each position *Ai*, Score(*A*_*i*_), is calculated as follows:

$$Score(A_{i})=\sum_{j=-10}^{+10}\chi^{\text{2}}{\text{(A}}_{\text{i}}{\text{,A}}_{\text{j}}\text{)},j\neq i$$ (2)

The position *Ai* with a maximal dependence value, Score(Ai), is applied to cluster sequences. When applying MDD to cluster sequences, a parameter, i.e., the maximum-cluster-size, should be set. If the size of a subgroup is less than the cutoff value of maximum-cluster-size, the subgroup will not be divided any further. The MDD process terminates after all of the subgroup sizes are less than the value of the specified maximum-cluster-size.

MDD clustering is a recursive process that divides all sequences into tree-like subgroups. After the detection of maximal dependence of flanking positions, as the example illustrated in Fig 1B, position +8 had the maximal dependence with the occurrence of basic amino acids. Subsequently, all data can be divided into two subgroups: one had the occurrence of basic amino acids in position +8 and the other lacked the occurrence of basic amino acids in position +8. The MDD clustering was a recursively process to divide the positive sets into tree-like subgroups. When applying MDDLogo to cluster the sequences of a positive set, a parameter, i.e., the maximum-cluster-size, should be set. If the size of a subgroup was less than the maximum-cluster-size, the subgroup will not be divided any more. In order to obtain an optimal minimum cluster size, MDDLogo was executed using various values. For this investigation, each subgroup resulting from MDDLogo was represented using WebLogo [24] for determining if they presented conserved motifs for the substrate specificity of *S*-glutathionylation.

### Model construction and evaluation

The support vector machine (SVM) was adopted to learn the predictive model from the positive and negative data of the training set. Based on binary classification, the concept behind SVM was to map the input samples into a higher dimensional space using a kernel function, followed by finding a hyper-plane that can discriminating the two classes with maximal margin and minimal error. A public SVM library, LIBSVM [41], was employed to generate the predictive models trained with various features. The radial basis function (RBF) *K*(*S*_*i*_, *S*_*j*_) = exp(−*γ*||*S*_*i*_ − *S*_*j*_||^2^) was used as the kernel function of the SVMs. The LIBSVM library could output a value of probability estimated ranging from 0 to 1 for each prediction. According to that, the values of probability estimated from each SVM classifier trained with the best feature corresponding to a specific motif were adopted to form an input vector for second-layered SVM.

**Fig 2:**
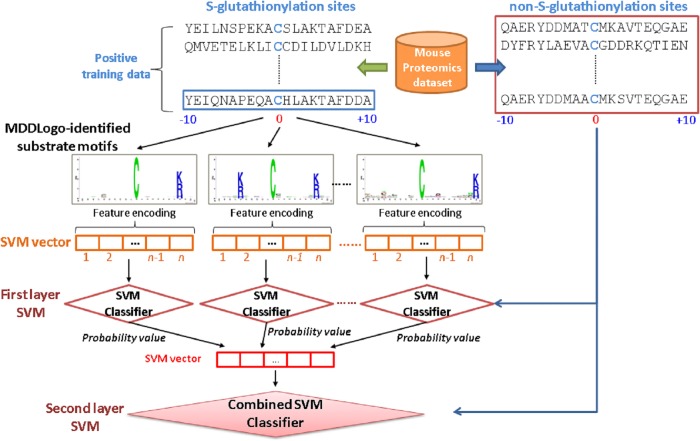
The conceptual diagram of two-layered SVMs trained with MDDLogo- identified substrate motifs.

Prior to the construction of a final model, the predictive performance of models using different features was evaluated by performing five-fold cross validation. As shown in Fig 2, firstly, the training data was divided into five groups by splitting each dataset into five approximately equal sized subgroups. During cross-validation, one subgroup was regarded as the test set, and the remaining four subgroups were regarded as the training set. The cross-validation process was repeated five times, in which each subgroup was used as a test set once. The five validation results were then combined to produce a single estimation. The advantage of cross-validation evaluation was that all original data were regarded as both training set and testing set, and each data was used for testing exactly once [42]. The following measures were then used to gauge the predictive performance of the trained models:

Sensitivity(Sn)=TP/(TP+FN) (3)

Specificity(Sp)=TN/(TN+FP) (4)

Accuracy(Acc)=(TP+TN)/(TP+FP+TN+FN) (5)

$$\text{Matthews Correlation Coefficient}\;\left(\text{MCC}\right)=\frac{\left(TP\text{×}TN\right)-\left(FN\text{×}FP\right)}{\sqrt{\left(TP+FN\right)\text{×}\left(TN+FP\right)\text{×}\left(TP+FP\right)\text{×}\left(TN+FN\right)}}$$ (6)

where TP, TN, FP and FN represented the numbers of true positives, true negatives, false positives and false negatives, respectively. After the selection of the predictive model with best performance, an independent testing was carried out to further evaluate the predictive performance of the best model (two-layered SVM).

## Results and Discussion

### Positively charged and higher solvent accessible amino acids neighboring with the *S*-glutathionylation site

**Fig 3:**
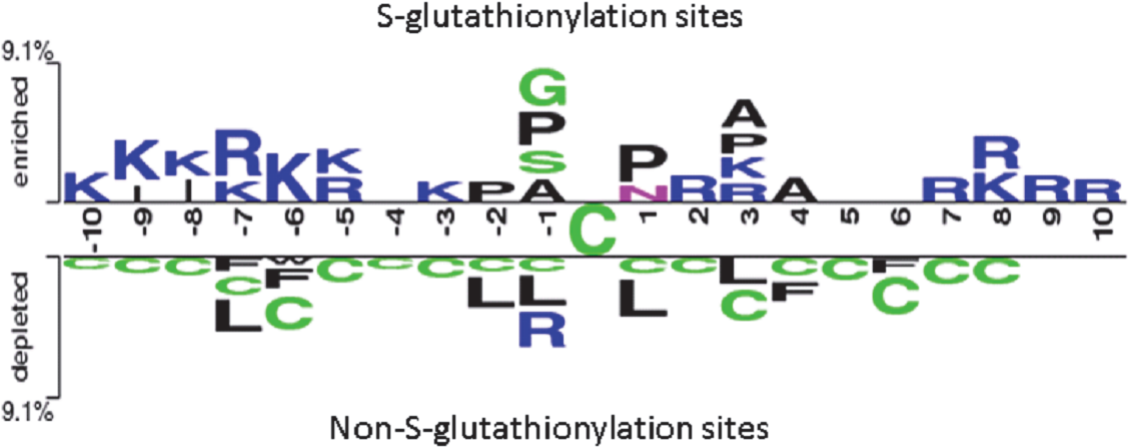
TwoSampleLogo presents the compositional biases of amino acids around *S*-glutathionylation sites compared to the non-*S*-glutathionylation sites in mouse macrophages. The significant amino acids around *S*-glutathionylated cysteine residue is enriched from the positive dataset and presented in upper panel (*p* < 0.01). Relatively, the high frequency of amino acids around non-*S*-glutathionylated cysteines is depleted from the negative dataset and presented in lower panel.

To explore the potential consensus motifs of *S*-glutathionylation, in this study, we focused on the sequence-based analysis of substrate specificity for *S*-glutathionylation. Here, a web-based tool TwoSampleLogo [43], that detected and displayed statistically significant differences in position-specific symbol compositions between two sets of multiple sequence alignments, was applied. In the preliminarily evaluation of the amino acid frequency neighboring the *S*-glutathionylated cysteine, the non-homologous *S*-glutathionylated cysteines were centered on position 0, and the flanking amino acids (-10 ~ +10) were graphically visualized as sequence logos. Contrast between 1783 *S*-glutathionylation sites and 8423 non-*S*-glutathionylation sites, the TwoSampleLogo revealed that the most pronounced feature of *S*-glutathionylation sites was the abundance of charged amino acids, especially the positively charged Lysine (K) and Arginine (R), at positions -10 ~ -5, -3, +2, +3, and +7 ~ +10 (*p* < 0.01, Fig 3, upper panel). Another interesting feature was the absence of positively charged residues at position -2, -1, +1, and +4 that was immediately adjacent to the *S*-glutathionylation sites. Comparatively, another featured characteristic, such as neutral amino acids Leucine (L), Phenylalanine (F), and Tryptophan (W), locating around non-*S*-glutathionylated cysteines at position -7, -6, -2, -1, +1, +3, +4, and +6, was depleted in the negative dataset (Fig 3, lower panel). Cysteine (C) residues also randomly located around non-*S*-glutathionylated cysteines from -10 ~ +8. This investigation also implicated that the notable difference of amino acid characteristic in sequence located around position -7, -6, -1, and +3. The analysis also revealed that the distant amino acids in sequence had significant difference between *S*-glutathionylation sites and non-*S*-glutathionylation sites, indicating that positively charged amino acids may be close to *S*-glutathionylated cysteines in three-dimensional structure.

In addition to the composition of amino acids in linear sequences, we further used RVP-Net algorithm to analyze the correlation of *S*-glutathionylation sites and solvent **accessible surface area** (ASA). As shown in S2 Fig, the comparison of average percentage of ASA in the 21-mer window (-10 ~ +10) showed that the cysteine residues had the lowest ASA on both *S*-glutathionylated and non-*S*-glutathionylated cysteines, suggesting low preference of solvent accessibility in *S*-glutathionylation sites. Moreover, the adjacent amino acids neighboring the centered *S*-glutathionylation sites had relatively higher preference of solvent-accessible surface area than that of non-*S*-glutathionylation sites. The result suggested that the flanking amino acids having hydrophilic characteristics may regulate the *S*-glutathionylation on cysteine residues due to the relative surface solvent accessibility.

### Cross-validation performance of training features

To determine what features provide the best performance to identify the *S*-glutathionylation sites compared to the non-*S*-glutathionylation sites, the predictive models were trained with various features, such as 20D binary code, BLOSUM62, AAC, AAPC, ASA, PWM, and PSSM. Using cross validation, four predictive powers, including sensitivity (Sn), specificity (Sp), accuracy (Acc), and Matthews correlation coefficient (MCC), were also evaluated. As shown in Table 2, the SVM models that were trained with 20D binary code generated the predictive sensitivity, specificity, accuracy, and MCC at 0.63, 0.63, 0.63, and 0.20, respectively. The similar quality was presented from that analyzed by BLOSUM62, AAC, AAPC, and PSSM. However, the model trained with ASA and PWM had the lowest predictive accuracy at 0.57 and relatively lower sensitivity, specificity, and MCC at 0.55, 0.57, and 0.1, respectively, which was probably caused by the low ASA value of cysteines. The specificity of the model trained with AAPC was equal to that with BLOSUM62 at 0.66 and slightly superior to that with other features. Given that AAPC was regarded as the best feature for training a model for discrimination of 1783 *S*-glutathionylation sites, the predictive sensitivity, specificity, accuracy, and MCC of the best model were 0.65, 0.66, 0.66, and 0.24, respectively. Additionally, the predictive power of the model trained with the hybrid combination of BLOSUM62 and AAPC provided the best performance of the predictive sensitivity, specificity, accuracy, and MCC at 0.66, 0.67, 0.67, and 0.26, respectively. Thus, BLOSUM62 combined with AAPC was selected as the training feature for the construction of two-layered SVM model.

**Table 2:** Five-fold cross validation results on single SVM model trained with various features.

**Training features**	**Sn**	**Sp**	**Acc**	**MCC**
20D Binary code	0.63	0.63	0.63	0.20
BLOSUM62	0.63	0.66	0.65	0.22
Amino Acid Composition (AAC)	0.63	0.65	0.65	0.22
Amino Acid Pair Composition (AAPC)	0.65	0.66	0.66	0.24
Accessible Surface Area (ASA)	0.55	0.57	0.57	0.10
Position Weight Matrix (PWM)	0.57	0.58	0.57	0.11
Position-specific scoring matrix (PSSM)	0.64	0.65	0.65	0.22
BLOSUM62 + AAPC	0.66	0.67	0.67	0.26

Total 1783 cysteine sequences were applied in positive and negative data. Sn, sensitivity; Sp, specificity; Acc, accuracy; MCC, Matthews Correlation Coefficient.

### MDD-clustered substrate motifs and the cross-validation performances

**Fig 4:**
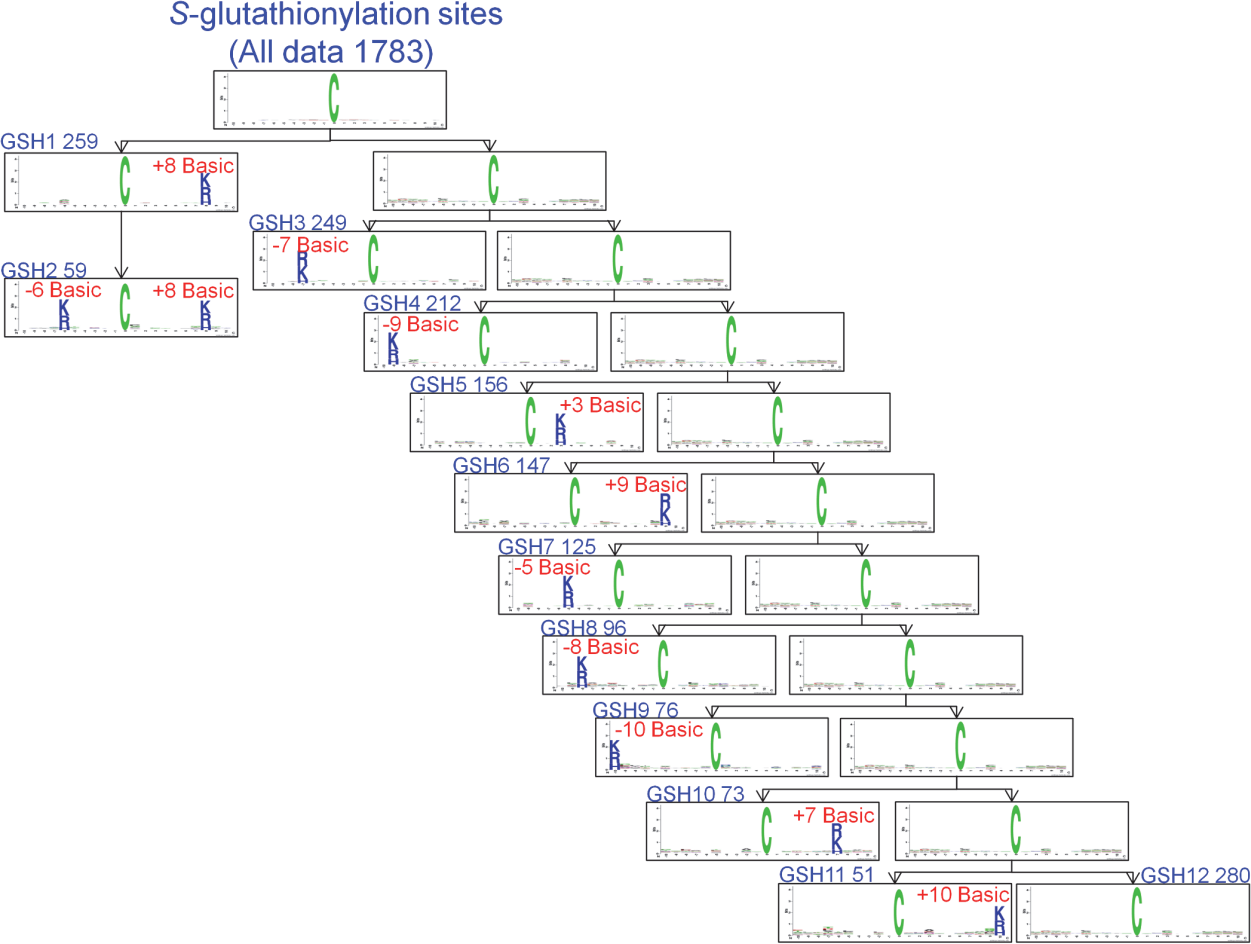
The MDDLogo-clustered subgroups from 1783 *S*-glutathionylation sites in mouse data set.

To improve the detection of the conserved motifs from large-scale *S*-glutathionylation data set, we further applied the maximal dependence decomposition (MDD) to cluster all 1783 identified *S*-glutathionylated peptide sequences. Here, 12 subgroups of *S*-glutathionylation motifs can be obtained from the most significant dependencies of amino acid composition between specific positions (Table 3 and Fig 4). According to the chi-square test of the dependence of five amino acid groups in flanking positions, 11 out of all MDDLogo-clustered subgroups had the conserved motifs of positively charged amino acids (K, R and H) at a specific position. In particular, 59 *S*-glutathionylated peptides in the second subgroup extracted from the 259 *S*-glutathionylated peptides in first subgroup had two positively charged amino acids on conserved motifs at two specific positions -6 and +8. Based on these significant frequency of sequence logos, the result was consistent with a previous data describing that the *S*-glutathionylated cysteine located in a basic environment perhaps in close three-dimensional proximity to K/R/H is more accessible [8]. However, the 12th subgroup containing the remaining 280 *S*-glutathionylation sites which did not have any conserved motif.

**Table 3:** The 12 MDDLogo-clustered subgroups and their performances of five-fold cross-validations from 1783 *S*-glutathionylation sites in mouse data set.

**MDDLogo Cluster**	**Number of positive data**	**C (cost)**	***γ* (gamma)**	**Sn**	**Sp**	**Acc**	**MCC**
GSH1	259	0.03125	0.0078125	0.67	0.69	0.68	0.28
GSH2	59	32768	0.5	0.71	0.76	0.75	0.39
GSH3	249	32768	0.5	0.69	0.70	0.70	0.30
GSH4	212	32768	0.5	0.68	0.69	0.69	0.29
GSH5	156	0.03125	0.0078125	0.65	0.66	0.66	0.21
GSH6	147	0.03125	0.0078125	0.70	0.71	0.71	0.33
GSH7	125	2048	8	0.72	0.75	0.74	0.37
GSH8	96	0.03125	0.0078125	0.69	0.70	0.70	0.31
GSH9	76	32768	0.5	0.74	0.80	0.79	0.45
GSH10	73	0.03125	0.0078125	0.66	0.68	0.68	0.27
GSH11	51	32768	0.5	0.67	0.71	0.70	0.29
GSH12	280	0.03125	0.0078125	0.65	0.69	0.68	0.26
All data	1783	0.03125	0.0078125	0.66	0.67	0.67	0.26
**Combined MDDLogo-clustered motifs**	**1783**	**0.03125**	**0.0078125**	**0.69**	**0.71**	**0.71**	**0.32**

C, cost value; *γ*, gamma value; Sn, sensitivity; Sp, specificity; Acc, accuracy; MCC, Matthews Correlation Coefficient.

Furthermore, we evaluated all of the *S*-glutathionylation sites and these 12 MDDLogo-clustered subgroups for their predictive performance by five-fold cross-validation. The predictive and average value of cross-validation performance in each subgroup was displayed in Table 3. Among them, subgroup GSH9, which had a conserved K/R/H at position -10, contained the highest predictive power at 0.74, 0.80, 0.79, and 0.45 for sensitivity, specificity, accuracy, and MCC, respectively. Moreover, the subgroup GSH2 presenting a conserved K/R/H at position -6 and +8 yielded the next best specificity, accuracy, and MCC at 0.76, 0.75, and 0.39. The predictive performance in all of these 12 subgroups of MDDLogo-clustered SVMs was presented higher sensitivity, specificity, accuracy, and MCC than that of all 1783 *S*-glutathionylation sites without any clustering. On the other hand, the SVM model trained with the combined MDDLogo-clustered motifs generated an enhanced performance of sensitivity, specificity, accuracy, and MCC at 0.69, 0.71, 0.71, and 0.32, compared with all 1783 *S*-glutathionylation sites without any clustering which contained lower performance at 0.65, 0.66, 0.66, and 0.24, respectively. This analysis indicated that the *S*-glutathionylated sequences in a large-scale data set can be alternatively clustered by MDD method, which significantly enhanced the signal of amino acids motif and improved the performance of the predictive model. Thus, the two-layered SVM model combining all MDDLogo-identified substrate motifs was utilized to implement a web-based prediction tool in website.

### Evaluation of *S*-glutathionylation predictive models using independent test set

**Fig 5:**
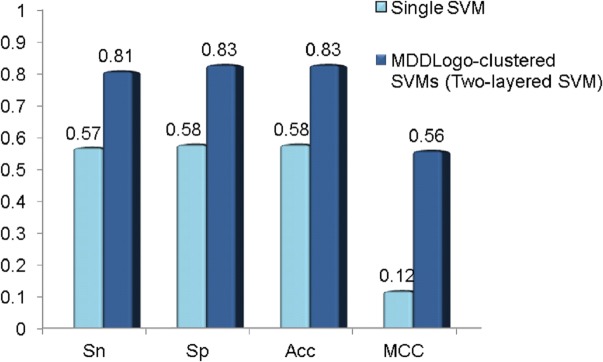
Comparison of independent testing performance between single SVM and MDDLogo-clustered SVM models. Sn, sensitivity; Sp, specificity; Acc, accuracy; MCC, Matthews Correlation Coefficient.

To evaluate effectiveness of the investigated features that achieved the best accuracy in cross-validation, an independent test set of *S*-glutathionylation was used to test the MDDLogo-clustered models training. The independent test set was composed of the experimentally verified *S*-glutathionylation data from multiple species, which contains a total of 254 positive data and 1054 negative data in 170 *S*-glutathionylated proteins. As shown in Fig 5, the MDD-clustered models could perform with a sensitivity of 0.57, a specificity of 0.58, an accuracy of 0.58, and the MCC of 0.12 in independent test set. Additionally, the two-layered SVM models using all the MDDLogo-clustered substrate motifs accomplished a sensitivity of 0.81, a specificity of 0.83, an accuracy of 0.83, and the MCC of 0.56. Overall, the independent testing demonstrated that the MDD-clustered models had higher estimated specificity comparing to sensitivity. Therefore, greater prediction power can be obtained by using MDDLogo-clustered SVM models than that by single SVM model. The detailed independent testing results were presented in S2 Table.

### Implementation of web-based tool for identification of *S*-glutathionylation sites

With the time-consuming and laboratory-intensive experimental workflow, even though a protein can be *S*-glutathionylated, precise identification of the *S*-glutathionylation sites on the substrate is still experimentally challenging. Therefore, developing an effective prediction tool can efficiently help identify potential *S*-glutathionylation sites. Following evaluation by cross-validation and an independent test, the MDDLogo-clustered models trained with combination of BLOSUM62 and AAPC are utilized in the construction of web-based prediction system, GSHsite. After the users submit their uncharacterized protein sequences, GSHSite efficiently returns the predictions including *S*-glutathionylated position, the flanking amino acids, and the matched MDDLogo-clustered motif. In addition, the protein sequences in FASTA format or protein name, gene name, and accession number can be used to search and predict. After submitting the information of peptide sequence or protein, the detail information and annotation of target protein, *S*-glutathionylation sites in published literature, and predicted *S*-glutathionylation motifs will be presented.

**Fig 6:**
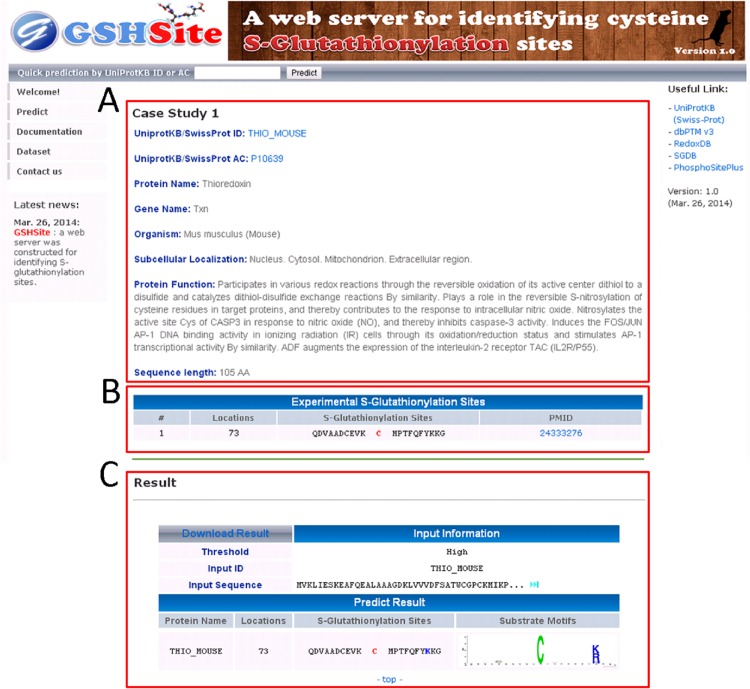
A case study of S-glutathionylation site prediction for mouse thioredoxin (THIO_MOUSE). The website presents (A) protein information and annotation, (B) S-glutathionylation sites from published experiment, and (C) prediction result of potential consensus motifs.

A mouse thioredoxin (TXN, THIO_MOUSE), which contains one *S*-glutathionylation site at Cys-73 [44], is used to demonstrate the effectiveness of GSHSite. As presented in Fig 6, GSHSite is able to correctly predict the experimentally verified *S*-glutathionylation site at positions 73. The matched MDD-clustered motif is also provided for the future investigation of substrate site specificity. The second case study was performed on human protein tyrosine phosphatase 1B (PTP1B, PTN1_HUMAN), which contains one *S*-glutathionylation site at cys-215 [45] and is not included in the training data set. The experimentally verified *S*-glutathionylation site at position 215 was also correctly predicted by GSHSite.

### Characteristic relationship between *S*-nitrosylation and *S*-glutathionylation

Because the *S*-nitrosylation and *S*-glutathionylation can target on the same cysteine residues of proteins, here, we further investigated the relationship between these two modifications. Based on the experimentally verified data set from dbSNO [46] and the study of Su. et al. [18], total 495 consistent sites from mouse were presented in total 1783 *S*-glutathionylation sites and total 2159 *S*-nitrosylation sites (S3 Table). We further analyzed the potential consensus motifs to explore the substrate specificity of these two modifications presenting by TwoSampleLogo. Contrast between 495 identically common sites and 1711 un-modified sites, the TwoSampleLogo revealed that the most pronounced features neighboring the modified sites were the abundance of the positively charged K/R at positions -7, +8, and +9, hydrophobic amino acids Isoleucine (I)/Proline (P) at position -6 ~ -4, +1, and +3, and polar amino acids Serine (S)/Threonine (T) at position -10, -1, +6 and +7 (*p* < 0.01, S3A Fig, upper panel). Comparatively, three amino acids, including hydrophobic amino acid Methionine (M), locating around un-modified cysteines at position -2, positively charged amino acid K at position -1, and polar amino acid Cysteine (C) randomly located from position -6 to +5, were depleted in the negative dataset (S3A Fig lower panel). This result implicated that the positively charged amino acids in distant sequence and hydrophobic amino acids surrounding *S*-glutathionylation and *S*-nitrosylation sites have notable difference of amino acid characteristics comparing with non-modification sites. For instance, the Cys73 on thioredoxin (Trx, THIO_MOUSE) in case 1 study was categorized into C(X)_7_K/R/H motif (GSH1 group in Table 3). Trx is one of the well-studied regulators to catalyze the transnitrosylation and denitrosylation of specific targets, including Cys215 on PTP1B (case 2 study, GSH3 group in Table 3), depending on the redox status of different cysteine residues [47–49]. In addition, Trx can also be *S*-glutathionylated at Cys73 and functioned as the deglutathionylase to regulate the enzymatic activity [50,51]. Due to the detail mechanism of *S*-nitrosylation and *S*-glutathionylation is still unclear *in vivo*, in this study, we proposed that the identified motifs for substrate specificity may help shed light to the study of the site-specific interplay between these two modifications.

After depleting the identically common cysteines, 1664 and 1288 cysteines were respectively presented in *S*-nitrosylation only and *S*-glutathionylation only (S4 Table). We further investigated the characteristic difference using TwoSampleLogo between these two modifications. This investigation also implicated that the positively charged amino acids R and polar amino acids Glutamine (Q), S, and C in sequence (around position -10 ~ -7, -4 ~ -1, +1 ~ +3, +8, and +10), had notable difference of amino acid characteristics between *S*-glutathionylation sites and *S*-nitrosylation sites (S3B Fig, upper panel). Comparatively, more hydrophobic amino acids in sequence had significant difference surrounding *S*-nitrosylation sites. The result indicated that polar and positively charged amino acids might be close to *S*-glutathionylated cysteines in three-dimensional structure.

To further understand the characteristics and categories of potential biological processes of these proteins, we also analyzed the annotation of Gene Ontology (GO) for cross talk of 328 *S*-glutathionylated and *S*-nitrosylated proteins by DAVID software (p < 0.01). S5 Table showed that most identically common proteins modifying by *S*-glutathionylation and *S*-nitrosylation contributed for translation and generation of precursor metabolites and energy. Moreover, more proteins involving in structural constituent of ribosome, structural molecule activity, and nucleotide binding were presented. In addition to the mitochondrial proteins, most proteins were located in cytosol and ribonucleoprotein complex. Similar biological processes and molecular functions were presented in only *S*-glutathionylated proteins (S6 Table). For the *S*-nitrosylation only, 133 of 974 proteins (14%) involved in oxidation reduction, 246 proteins (25%) played roles in nucleotide binding, and 333 (34%) proteins located in mitochondrion (S7 Table).

## Conclusion

In this study, we reported a systematic informatics investigation on the *S*-glutathionylation substrate specificity from experimentally verified *S*-glutathionylomic data. The analysis of position-specific amino acids composition reveals that the most pronounced feature of *S*-glutathionylation sites is the abundance of positively charged amino acids at surrounding positions, especially on the positions from -5 to +3. This investigation also implicates that the distant amino acids in sequence (around position -7 and -6), which may be close to *S*-glutathionylation cysteines in three-dimensional structure, have notable difference between *S*-glutathionylation sites and non-*S*-glutathionylation sites. Moreover, the flanking amino acids around *S*-glutathionylation sites have higher preference of solvent-accessible surface area than that around non-*S*-glutathionylation sites. According to the five-fold cross-validation, the model trained with the combined features of BLOSUM62 and amino acid pair composition gets the highest sensitivity, specificity, accuracy, and MCC.

Due to the abundance of experimental data, this study focuses on investigating the motifs of *S*-glutathionylation sites based on the amino acid sequences. However, it is difficult to explore the conserved motifs from large-scale S-glutathionylome data set. Thus, this work applies MDDLogo algorithm to cluster all sequences of *S*-glutathionylation site into 12 subgroups. According to the chi-square test of the dependence in flanking positions, surprisingly, all of the MDD-clustered subgroups have the conserved motifs of positively charged amino acids (K, R and H) at a specific position. Particularly, subgroups GSH2 have the conserved motifs of positively charged amino acids at two specific positions (-6 and +8). Although the newly identified motifs could not be experimentally verified, it still worthy to be noticed that MDD clustering can help the biologist investigating the potential substrate motifs of *S*-glutathionylation sites. More noteworthy is that the MDD-clustered motifs can be applied to improve the predictive power of computationally identifying *S*-glutathionylation sites with various substrate specificities. According to the evaluation of five-fold cross-validation, the models trained with combined MDD-clustered motifs are increased for the predictive accuracy of 0.71, comparing to the model trained without MDD clustering. This analysis indicates that the *S*-glutathionylated sequences with a larger size can be alternatively clustered by MDD method in order to enhance the signal of amino acids motif and improve the performance of the predictive model.

Finally, the independent testing indicates that the predictive model by MDDLogo-clustered SVMs can generate the best performance compared with the single SVM model. The acquisition of additional experimentally verified *S*-glutathionylation data is needed to re-calibrate more accurate MDD-clustered motifs. The proposed method can be improved by considering the motifs that are intrinsically included in the test data. Consequently, the models with MDD clustering method are applied to implement a novel web-based tool, named GSHSite, for identifying cysteine *S*-glutathionylation. Correct prediction on two experimentally verified *S*-glutathionylated proteins demonstrated the effectiveness of GSHSite. In this web-based tool, the detail information, annotation, and 3D structure provided from PDB of proteins are also included. This approach not only provides the prediction yet experimental *S*-glutathionylation site information, but also can be used to explore the potential substrate specificity of *S*-glutathionylation.

## Availability

The proposed method is implemented as a web-based resource, which is now freely available to all interested users at http://csb.cse.yzu.edu.tw/GSHSite/. All of the data set used in this work is also available for download in the website.

## Supporting Information

S1 Fig The encoding scheme of the amino acid pair composition (AAPC) combined with BLOSUM62 feature. (TIF)

S2 Fig Comparison of solvent-accessible surface area between *S*-glutathionylation and non-*S*-glutathionylation sites. (TIF)

S3 Fig TwoSampleLogo presents the compositional biases of amino acids around *S*-glutathionylation sites compared to the *S*-nitrosylation sites in mouse dataset. (A) The identically common cysteines for *S*-glutathionylation and *S*-nitrosylation in upper panel were compared with un-modified cysteines in lower panel (p < 0.01). (B) The significant amino acids around *S*-glutathionylated cysteine residue were enriched from the positive dataset and presented in upper panel (*p* < 0.01). Relatively, the high frequency of amino acids around *S*-nitrosylated cysteines were depleted from the negative dataset and presented in lower panel.
(TIF)

S1 Table The amino acids group of MDDLogo used in this study. (DOCX)

S2 Table The detailed results of training and independent testing comparison between our method. (DOCX)

S3 Table The number of proteins and sites in each *S-*glutathionylation and *S-*nitrosylation data. (DOCX)

S4 Table The detail information in *S-*glutathionylation and *S-*nitrosylation data by two-layered SVMs analysis. (DOCX)

S5 Table The top 10 distributions of Gene Ontology (GO) annotations for cross talk of *S*-glutathionylated and *S*-nitrosylated proteins by DAVID analysis (p < 0.01). (DOCX)

S6 Table The top 10 distributions of GO annotations for only *S*-glutathionylated proteins by DAVID analysis (p < 0.01). (DOCX)

S7 Table The top 10 distributions of GO annotations for only *S*-nitrosylated proteins by DAVID analysis (p < 0.01). (DOCX)
