# Supplementary figures and images for: GSHSite: Exploiting an Iteratively Statistical Method to Identify S-Glutathionylation Sites with Substrate Specificity
Source: PLoS One. 2015 Apr 7;10(4):e0118752. doi: 10.1371/journal.pone.0118752 (PMC4388702; doi:10.1371/journal.pone.0118752)

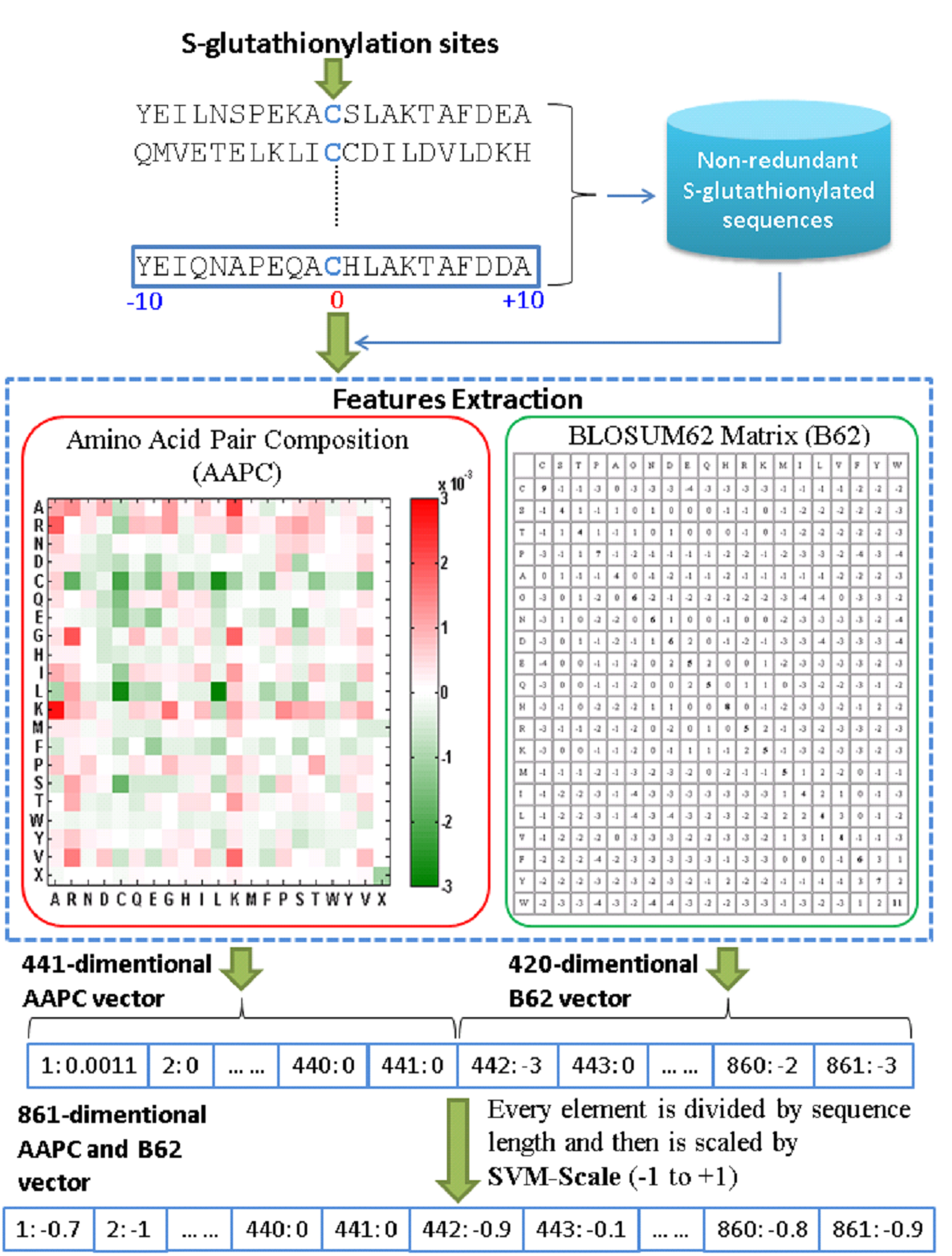

Supplement: S1 Fig — (TIF) [file pone.0118752.s001.tif]

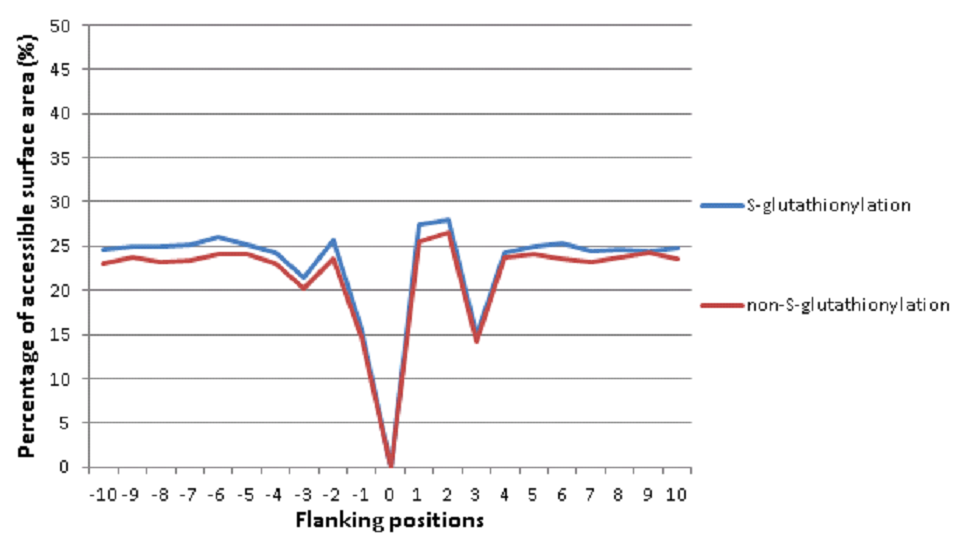

Supplement: S2 Fig — (TIF) [file pone.0118752.s002.tif]

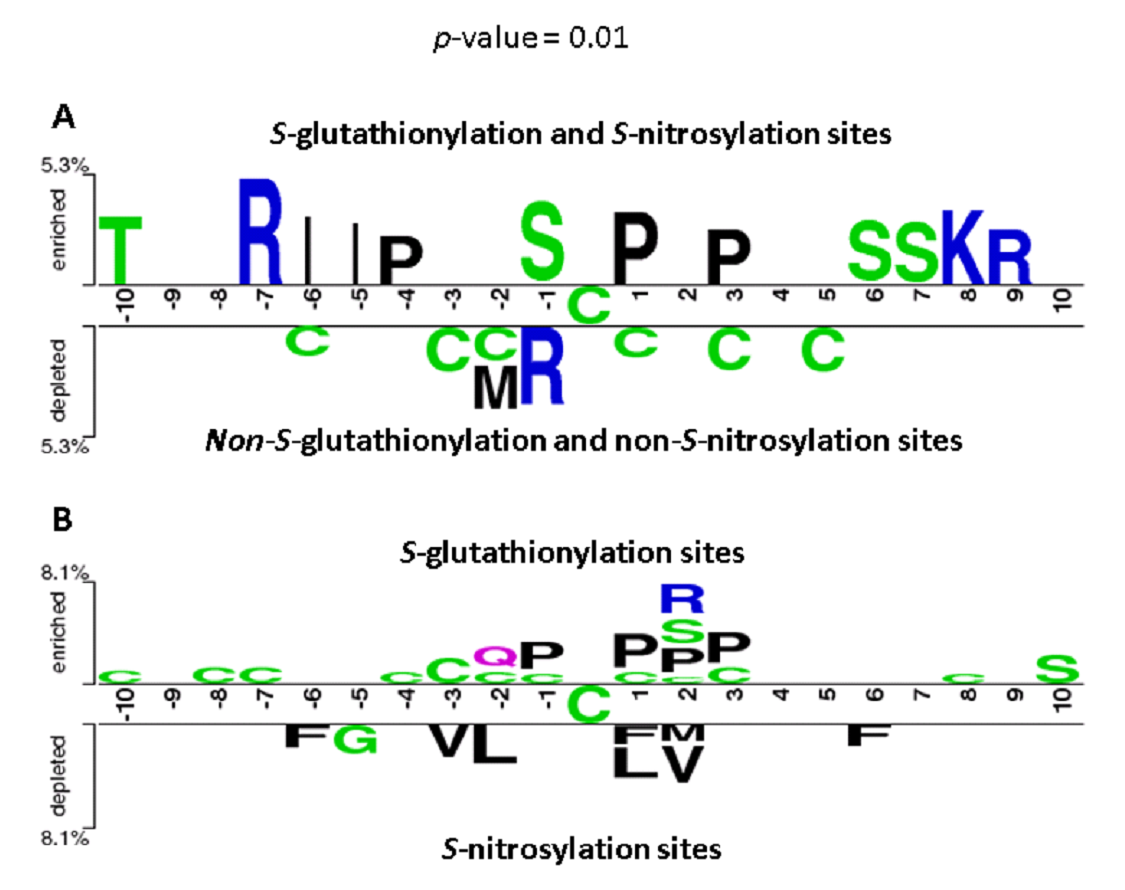

Supplement: S3 Fig — (A) The identically common cysteines for S-glutathionylation and S-nitrosylation in upper panel were compared with un-modified cysteines in lower panel (p < 0.01). (B) The significant amino acids around S-glutathionylated cysteine residue were enriched from the positive dataset and presented in upper panel (p < 0.01). Relatively, the high frequency of amino acids around S-nitrosylated cysteines were depleted from the negative dataset and presented in lower panel. (TIF) [file pone.0118752.s003.tif]
