# Supplementary material for: GSHSite: Exploiting an Iteratively Statistical Method to Identify S-Glutathionylation Sites with Substrate Specificity
Source: PLoS One. 2015 Apr 7;10(4):e0118752. doi: 10.1371/journal.pone.0118752 (PMC4388702; doi:10.1371/journal.pone.0118752)
Supplement: S1 Table — (DOCX) [file pone.0118752.s004.docx]

**Table S1. The amino acids group of MDDLogo used in this study.**

| **Chemical properties** | **Amino acids** |
| --- | --- |
| **Polar group** | Glycine (G), Serine (S), Threonine (T), Cysteine (C), Glutamine (Q), Asparagine (N) |
| **Acidic group** | Aspartic acid (D), Glutamic acid (E) |
| **Basic group** | Lysine (K), Arginine (R), Histidine (H) |
| **Hydrophobic group** | Alanine (A), Valine (V), Leucine (L), Isoleucine (I), Proline (P), Methionine (M) |
| **Aromatic group** | Phenylalanine (F), Tyrosine (Y), Tryptophan (W) |
