# Supplementary material for: GSHSite: Exploiting an Iteratively Statistical Method to Identify S-Glutathionylation Sites with Substrate Specificity
Source: PLoS One. 2015 Apr 7;10(4):e0118752. doi: 10.1371/journal.pone.0118752 (PMC4388702; doi:10.1371/journal.pone.0118752)
Supplement: S2 Table — (DOCX) [file pone.0118752.s005.docx]

**Table S2. The detailed results of training and independent testing comparison between our method.**

| Data | Predictive model | TP | FP | TN | FN | Sn | Sp | Acc | MCC |
| --- | --- | --- | --- | --- | --- | --- | --- | --- | --- |
| Training | Single SVM | 1177 | 2779 | 5644 | 606 | 0.66 | 0.67 | 0.67 | 0.26 |
|  | Two-layered SVMs | 1230 | 2443 | 5980 | 553 | 0.69 | 0.71 | 0.71 | 0.32 |
| Independent testing | Single SVM | 144 | 440 | 614 | 110 | 0.57 | 0.58 | 0.58 | 0.12 |
|  | Two-layered SVMs | 206 | 177 | 877 | 48 | 0.81 | 0.83 | 0.83 | 0.56 |
