# Supplementary material for: GSHSite: Exploiting an Iteratively Statistical Method to Identify S-Glutathionylation Sites with Substrate Specificity
Source: PLoS One. 2015 Apr 7;10(4):e0118752. doi: 10.1371/journal.pone.0118752 (PMC4388702; doi:10.1371/journal.pone.0118752)
Supplement: S3 Table — (DOCX) [file pone.0118752.s006.docx]

**Table S3. The number of proteins and sites in each *S-*glutathionylation and *S-*nitrosylation data.**

| **Dataset** | **Number of proteins** | **Number of sites** |
| --- | --- | --- |
| All *S-*glutathionylation data  (Training data) | 1003 | 1783 |
| All *S*-nitrosylation data  (dbSNO mouse data) | 1156 | 2159 |
| Cross talk of *S*-glutathionylation and *S*-nitrosylation data | 328 | 495 |
| Only *S*-glutathionylation data | 807 (575)* | 1288 |
| Only *S*-nitrosylation data | 974 (740)* | 1664 |

*, depletion of the same proteins without the consistent modification site.
