# Supplementary material for: GSHSite: Exploiting an Iteratively Statistical Method to Identify S-Glutathionylation Sites with Substrate Specificity
Source: PLoS One. 2015 Apr 7;10(4):e0118752. doi: 10.1371/journal.pone.0118752 (PMC4388702; doi:10.1371/journal.pone.0118752)
Supplement: S4 Table — (DOCX) [file pone.0118752.s007.docx]

**Table S4. The detail information in *S-*glutathionylation and *S-*nitrosylation data by two-layered SVMs analysis.**

| **Dataset** | **Number of sites** | **TP** | **FP** | **TN** | **FN** | **Sn** | **Sp** | **Acc** | **MCC** |
| --- | --- | --- | --- | --- | --- | --- | --- | --- | --- |
| Only *S*-glutathionylation data (Positive) | 1288 | 780 | 665 | 999 | 508 | 0.60 | 0.60 | 0.60 | 0.20 |
| Only *S*-nitrosylation data (Negative) | 1664 |  |  |  |  |  |  |  |  |
