# Supplementary material for: GSHSite: Exploiting an Iteratively Statistical Method to Identify S-Glutathionylation Sites with Substrate Specificity
Source: PLoS One. 2015 Apr 7;10(4):e0118752. doi: 10.1371/journal.pone.0118752 (PMC4388702; doi:10.1371/journal.pone.0118752)
Supplement: S5 Table — (DOCX) [file pone.0118752.s008.docx]

**Table S5. The top 10 distributions of Gene Ontology (GO) annotations for cross talk of *S*-glutathionylated and *S*-nitrosylated proteins by DAVID analysis (p < 0.01).**

| **GO ID** | **GO Terms** | **Number of proteins** | **Total**  **(%)** | **P-Value** |
| --- | --- | --- | --- | --- |
| **GO Biological Processes** | | | |  |
| GO:0006412 | translation | 53 | 16.2 | 6.36E-32 |
| GO:0006091 | generation of precursor metabolites and energy | 36 | 11.0 | 8.35E-19 |
| GO:0045333 | cellular respiration | 16 | 4.9 | 7.29E-13 |
| GO:0006007 | glucose catabolic process | 15 | 4.6 | 1.83E-12 |
| GO:0019320 | hexose catabolic process | 15 | 4.6 | 1.83E-12 |
| GO:0009060 | aerobic respiration | 12 | 3.7 | 2.56E-12 |
| GO:0046365 | monosaccharide catabolic process | 15 | 4.6 | 3.23E-12 |
| GO:0044275 | cellular carbohydrate catabolic process | 15 | 4.6 | 1.54E-11 |
| GO:0046164 | alcohol catabolic process | 15 | 4.6 | 4.94E-11 |
| GO:0006096 | glycolysis | 13 | 4.0 | 6.15E-11 |
| **GO Molecular Function** | | | |  |
| GO:0003735 | structural constituent of ribosome | 35 | 10.7 | 1.20E-24 |
| GO:0005198 | structural molecule activity | 44 | 13.5 | 7.49E-16 |
| GO:0000166 | nucleotide binding | 101 | 30.9 | 3.88E-13 |
| GO:0017076 | purine nucleotide binding | 76 | 23.2 | 3.18E-07 |
| GO:0051082 | unfolded protein binding | 12 | 3.7 | 4.42E-07 |
| GO:0003723 | RNA binding | 38 | 11.6 | 4.94E-07 |
| GO:0032555 | purine ribonucleotide binding | 72 | 22.0 | 1.23E-06 |
| GO:0032553 | ribonucleotide binding | 72 | 22.0 | 1.23E-06 |
| GO:0070003 | threonine-type peptidase activity | 7 | 2.1 | 5.24E-06 |
| GO:0004298 | threonine-type endopeptidase activity | 7 | 2.1 | 5.24E-06 |
| **GO Cellular Component** | | | |  |
| GO:0005739 | mitochondrion | 94 | 28.7 | 2.46E-28 |
| GO:0005829 | cytosol | 59 | 18.0 | 8.51E-26 |
| GO:0030529 | ribonucleoprotein complex | 51 | 15.6 | 1.13E-22 |
| GO:0005840 | ribosome | 35 | 10.7 | 1.94E-22 |
| GO:0044429 | mitochondrial part | 43 | 13.1 | 2.54E-14 |
| GO:0031967 | organelle envelope | 40 | 12.2 | 6.29E-12 |
| GO:0031975 | envelope | 40 | 12.2 | 7.07E-12 |
| GO:0019866 | organelle inner membrane | 30 | 9.2 | 1.02E-11 |
| GO:0005743 | mitochondrial inner membrane | 28 | 8.6 | 8.53E-11 |
| GO:0031966 | mitochondrial membrane | 31 | 9.5 | 1.17E-10 |
