# Supplementary material for: GSHSite: Exploiting an Iteratively Statistical Method to Identify S-Glutathionylation Sites with Substrate Specificity
Source: PLoS One. 2015 Apr 7;10(4):e0118752. doi: 10.1371/journal.pone.0118752 (PMC4388702; doi:10.1371/journal.pone.0118752)
Supplement: S6 Table — (DOCX) [file pone.0118752.s009.docx]

**Table S6. The top 10 distributions of GO annotations for only *S*-glutathionylated proteins by DAVID analysis (p < 0.01).**

| **GO ID** | **GO Terms** | | **Number of proteins** | **Total**  **(%)** | **P-Value** |
| --- | --- | --- | --- | --- | --- |
| **GO Biological Processes** | | | | |  |
| GO:0006412 | translation | | 86 | 10.70 | 3.14E-41 |
| GO:0006418 | tRNA aminoacylation for protein translation | | 20 | 2.5 | 5.93E-14 |
| GO:0043039 | tRNA aminoacylation | | 20 | 2.5 | 5.93E-14 |
| GO:0043038 | amino acid activation | | 20 | 2.5 | 5.93E-14 |
| GO:0006413 | translational initiation | | 15 | 1.9 | 7.77E-10 |
| GO:0006399 | tRNA metabolic process | | 24 | 3.0 | 2.17E-09 |
| GO:0009161 | ribonucleoside monophosphate metabolic process | | 11 | 1.4 | 3.15E-09 |
| GO:0009167 | purine ribonucleoside monophosphate metabolic process | | 10 | 1.2 | 4.19E-09 |
| GO:0009126 | purine nucleoside monophosphate metabolic process | | 10 | 1.2 | 4.19E-09 |
| GO:0009156 | ribonucleoside monophosphate biosynthetic process | | 10 | 1.2 | 1.87E-08 |
| **GO Molecular Function** | | | | |  |
| GO:0000166 | nucleotide binding | | 204 | 25.4 | 1.38E-22 |
| GO:0003735 | structural constituent of ribosome | | 38 | 4.7 | 1.01E-16 |
| GO:0017076 | purine nucleotide binding | | 169 | 21.0 | 1.92E-16 |
| GO:0032553 | ribonucleotide binding | | 163 | 20.2 | 4.51E-16 |
| GO:0032555 | purine ribonucleotide binding | | 163 | 20.3 | 4.51E-16 |
| GO:0030554 | adenyl nucleotide binding | | 144 | 17.9 | 2.79E-15 |
| GO:0003723 | RNA binding | | 83 | 10.3 | 5.09E-15 |
| GO:0001883 | purine nucleoside binding | | 144 | 17.9 | 5.58E-15 |
| GO:0032559 | adenyl ribonucleotide binding | | 138 | 17.2 | 7.03E-15 |
| GO:0001882 | nucleoside binding | | 144 | 17.9 | 9.33E-15 |
| **GO Cellular Component** | | | | |  |
| GO:0005829 | cytosol | | 87 | 10.8 | 1.18E-26 |
| GO:0030529 | ribonucleoprotein complex | | 68 | 8.5 | 5.31E-19 |
| GO:0048770 | pigment granule | | 29 | 3.6 | 5.88E-18 |
| GO:0042470 | melanosome | | 29 | 3.6 | 5.88E-18 |
| GO:0005840 | ribosome | | 41 | 5.1 | 3.49E-17 |
| GO:0000502 | proteasome complex | | 21 | 2.6 | 1.34E-13 |
| GO:0031988 | membrane-bounded vesicle | | 53 | 6.6 | 4.49E-12 |
| GO:0016023 | cytoplasmic membrane-bounded vesicle | | 52 | 6.5 | 8.88E-12 |
| GO:0031982 | vesicle | | 58 | 7.2 | 5.36E-11 |
| GO:0031410 | cytoplasmic vesicle | 56 | | 7.0 | 2.05E-10 |
