# Supplementary material for: GSHSite: Exploiting an Iteratively Statistical Method to Identify S-Glutathionylation Sites with Substrate Specificity
Source: PLoS One. 2015 Apr 7;10(4):e0118752. doi: 10.1371/journal.pone.0118752 (PMC4388702; doi:10.1371/journal.pone.0118752)
Supplement: S7 Table — (DOCX) [file pone.0118752.s010.docx]

**Table S7. The top 10 distributions of GO annotations for only *S*-nitrosylated proteins by DAVID analysis (p < 0.01).**

| **GO ID** | **GO Terms** | **Number of proteins** | **Total**  **(%)** | **P-Value** |
| --- | --- | --- | --- | --- |
| **GO Biological Processes** | | | |  |
| GO:0055114 | oxidation reduction | 133 | 13.9 | 3.96E-40 |
| GO:0006091 | generation of precursor metabolites and energy | 80 | 8.4 | 8.04E-38 |
| GO:0051186 | cofactor metabolic process | 50 | 5.2 | 2.50E-21 |
| GO:0006732 | coenzyme metabolic process | 44 | 4.6 | 6.74E-21 |
| GO:0006412 | translation | 66 | 6.9 | 8.98E-21 |
| GO:0045333 | cellular respiration | 29 | 3.0 | 3.33E-20 |
| GO:0044275 | cellular carbohydrate catabolic process | 28 | 2.9 | 8.93E-19 |
| GO:0015980 | energy derivation by oxidation of organic compounds | 34 | 3.6 | 5.23E-18 |
| GO:0046164 | alcohol catabolic process | 28 | 2.9 | 1.12E-17 |
| GO:0019320 | hexose catabolic process | 25 | 2.6 | 3.75E-17 |
| **GO Molecular Function** | | | |  |
| GO:0000166 | nucleotide binding | 246 | 25.7 | 1.75E-27 |
| GO:0048037 | cofactor binding | 64 | 6.7 | 1.26E-26 |
| GO:0050662 | coenzyme binding | 50 | 5.2 | 6.37E-23 |
| GO:0017076 | purine nucleotide binding | 194 | 20.3 | 5.68E-17 |
| GO:0032555 | purine ribonucleotide binding | 176 | 18.4 | 9.20E-13 |
| GO:0032553 | ribonucleotide binding | 176 | 18.4 | 9.20E-13 |
| GO:0001883 | purine nucleoside binding | 154 | 16.1 | 1.29E-11 |
| GO:0030554 | adenyl nucleotide binding | 153 | 16.0 | 1.31E-11 |
| GO:0001882 | nucleoside binding | 154 | 16.1 | 2.09E-11 |
| GO:0051287 | NAD or NADH binding | 17 | 1.8 | 1.37E-09 |
| **GO Cellular Component** | | | |  |
| GO:0005739 | mitochondrion | 333 | 34.8 | 2.53E-140 |
| GO:0044429 | mitochondrial part | 167 | 17.5 | 1.67E-80 |
| GO:0005740 | mitochondrial envelope | 113 | 11.8 | 1.91E-48 |
| GO:0031980 | mitochondrial lumen | 75 | 7.8 | 4.36E-48 |
| GO:0005759 | mitochondrial matrix | 75 | 7.8 | 4.36E-48 |
| GO:0031966 | mitochondrial membrane | 109 | 11.4 | 8.15E-48 |
| GO:0005743 | mitochondrial inner membrane | 93 | 9.7 | 5.57E-43 |
| GO:0019866 | organelle inner membrane | 94 | 9.8 | 9.76E-42 |
| GO:0031967 | organelle envelope | 124 | 13.0 | 1.06E-41 |
| GO:0031975 | envelope | 124 | 13.0 | 1.60E-41 |
